# Effects of mushroom and chicory extracts on the shape, physiology and proteome of the cariogenic bacterium *Streptococcus mutans*

**DOI:** 10.1186/1472-6882-13-117

**Published:** 2013-05-29

**Authors:** Caterina Signoretto, Anna Marchi, Anna Bertoncelli, Gloria Burlacchini, Alberto Milli, Francesco Tessarolo, Iole Caola, Adele Papetti, Carla Pruzzo, Egija Zaura, Peter Lingström, Itzhak Ofek, David A Spratt, Jonathan Pratten, Michael Wilson, Pietro Canepari

**Affiliations:** 1 Dipartimento di Patologia e Diagnostica – Sezione di Microbiologia, Università di Verona, Strada Le Grazie 8, Verona, 37134, Italy; 2 Centro Interdipartimentale di Tecnologie Biomediche (BIOtech), Università di Trento, Via delle Regole 101, Mattarello, Trento, 38123, Italy; 3 Sezione di Microscopia Elettronica, Dipartimento di Medicina di Laboratorio, APSS, Trento, 38100, Italy; 4 Dipartimento di Scienze del Farmaco, Università di Pavia, Via Taramelli 12, Pavia, 27100, Italy; 5 DISTAV, Università di Genova, Corso Europa 26, Genova, 16132, Italy; 6 Department of Preventive Dentistry, Academic Centre for Dentistry Amsterdam (ACTA), Gustav Mahlerlaan 3004, Amsterdam, LA, 1081, The Netherlands; 7 Department of Cariology, Institute of Odontology The Sahlgrenska Academy, University of Gothenburg, Box 450, Gothenburg, 405 30, Sweden; 8 Department of Clinical Microbiology and Immunology, Sackler Faculty of Medicine, Tel Aviv University, Tel Aviv, 39987, Israel; 9 Department of Microbial Diseases, UCL Eastman Dental Institute, 256 Gray’s Inn Road, London, WC1X 8LD, UK

**Keywords:** *Streptococcus mutans*, Dental caries, Anticaries compounds, Food components

## Abstract

**Background:**

Dental caries is an infectious disease which results from the acidic demineralisation of the tooth enamel and dentine as a consequence of the dental plaque (a microbial biofilm) accumulation. Research showed that several foods contain some components with antibacterial and antiplaque activity. Previous studies indicated antimicrobial and antiplaque activities in a low-molecular-mass (LMM) fraction of extracts from either an edible mushroom (*Lentinus edodes*) or from Italian red chicory (*Cichorium intybus*).

**Methods:**

We have evaluated the antimicrobial mode of action of these fractions on *Streptococcus mutans*, the etiological agent of human dental caries. The effects on shape, macromolecular syntheses and cell proteome were analysed.

**Results:**

The best antimicrobial activity has been displayed by the LMM mushroom extract with a bacteriostatic effect. At the MIC of both extracts DNA synthesis was the main macromolecular synthesis inhibited, RNA synthesis was less inhibited than that of DNA and protein synthesis was inhibited only by roughly 50%. The partial inhibition of protein synthesis is compatible with the observed significant increase in cell mass. The increase in these parameters is linked to the morphological alteration with transition from cocci of the untreated control to elongated cells. Interestingly, these modifications were also observed at sub-MIC concentrations. Finally, membrane and cytosol proteome analysis was conducted under LMM mushroom extract treatment in comparison with untreated *S*. *mutans* cells. Significant changes were observed for 31 membrane proteins and 20 of the cytosol fractions. The possible role of the changed proteins is discussed.

**Conclusions:**

This report has shown an antibiotic-like mode of action of mushroom and chicory extracts as demonstrated by induced morphogenetic effects and inhibition of specific macromolecular synthesis. This feature as well as the safe use of this extract as result of its natural origin render the LMM both mushroom and chicory extracts suitable for the formulation into products for daily oral hygiene such as mouthwashes or toothpastes.

## Background

Dental caries is an infectious disease of worldwide importance [1,2]. It results from the acidic demineralisation of the tooth enamel and dentine as a consequence of the dental plaque (a microbial biofilm) accumulation [3,4]. *Streptococcus mutans* and *Streptococcus sobrinus* are considered the main etiological agents of human dental caries due to their efficient colonization of the dental surface, efficient carbohydrate metabolism and lactic acid production (acidogenicity) and aciduricity (i.e. the capability to survive and multiply in acidic environment) [1,2]. Although cariogenic streptococci are included among the normal oral microbiota of humans, both diet and inefficient oral hygiene are the trigger for disease initiation and progression [3]. Tooth colonization by *S*. *mutans* is conventionally subdivided in a sucrose-independent and a sucrose-dependent phase. Initially, several adhesins of the odontopathogenic bacteria interact with the salivary glycoproteins of the acquired pellicle on teeth surface via bivalent cations (mainly Ca^++^). During the second phase bacteria adhere tightly to tooth surface as a result of the production of exopolysaccharides (glucans) from sucrose by the activity of distinct glucosyl transferases (GTFs) which act sequentially to produce a final, very hydrophobic, glucan called “mutan”. This is responsible for the final tight adhesion of bacteria to tooth surface. If dental plaque is allowed to accumulate, mutans streptococci efficiently metabolize sucrose (or sugar polymers such as starch) to produce large amount of lactic acid capable of solubilising the mineral component of the tooth and to initiate the carious process [1,2]. Several preventive strategies can be used to inhibit or slow down the cariogenic process. These range from fluoride administration, typically in toothpastes to strengthen the enamel and dentine (by substituting Cl^-^ for F^-^) to better resist the acid damage, to reduction in sugar diet or use of sugar substitutes (e.g. sorbitol and aspartame) [1,2]. However, it is generally accepted that lowering the oral bacterial count (i.e. dental plaque removal) is an effective method for preventing caries development. The manual dexterity of many individuals, however, is not very efficient and good oral hygiene is difficult to maintain. In order to compliment the mechanical nature of the cleaning mouthwashes and toothpastes which contain compounds with antimicrobial and antiplaque activities are recommended.

Research performed over the last three decades has shown that, contrary to the popular belief that foods have a negative impact on oral health, several foods contain some components with antibacterial and antiplaque activity for recent reviews see ref. [5,6]. It seems reasonable, thus, to encourage the consumption of such foods and/or incorporate the active compound(s) in functional foods or in cosmetic products for daily oral hygiene [7]. Furthermore, the trend for using “natural” compounds for oral health products instead of those obtained by chemical synthesis is gaining momentum. Previous studies have shown antimicrobial and antiplaque activities in a low-molecular-mass (LMM) fraction of extracts from either an edible mushroom (*Lentinus edodes*, very popular in Japan and called shiitake) or from Italian red chicory (early Treviso red chicory, an anthocyanic cultivar of *Cichorium intybus*) [8-10].

In this work we have evaluated in some detail the antimicrobial mode of action of the LMM fractions of mushroom and chicory aqueous extracts on *S*. *mutans*, the main etiological agent of human dental caries.

## Methods

### Bacterial strains and growth conditions

*S*. *mutans* UA159 was used throughout this study. Cells were grown in brain hearth infusion broth (BHIB) or on brain hearth infusion agar (BHIA) (Oxoid Ltd, Basingstoke, England). Cultures were incubated at 37°C in an atmosphere enriched with 5% CO_2_. Growth of *S*. *mutans* in a biofilm structure was allowed on ceramic hydroxyapatite (HA) discs (Clarkson Chromatography Products Inc., South Williamsport, PA, USA). To achieve this, HA discs (2.5 mm diameter for SEM or 25 mm for the other experiments) were coated with sterile human saliva collected from a pool of donors [11]. Each disc was then incubated in 5 ml of BHIB medium spread with a *S*. *mutans* culture in a well of a 6-well tissue culture plate (35 mm diameter, flat bottom, Sarstedt, Verona, Italy) at 37°C in the capnophylic atmosphere. Every day (for a total of three days) culture supernatants were removed by gentle aspiration and replaced with fresh medium. In order to analyse the effects of the test compound, on 3 day old biofilms a suitable concentration of LMM fraction of mushroom extract was added to the growth medium and incubated for additional 3 hours. At the end of each incubation, biofilms were washed three times with sterile distilled water and then fixed with 2.5% glutaraldehyde in 0.1 M phosphate buffer pH 7.2 for SEM analysis.

### LMM fraction of both mushroom and chicory extract

Frozen Shiitake mushroom was purchased from Asiago Food SpA, Veggiano, Padua, Italy, and red chicory from The Italian Consorzio Radicchio di Treviso, Treviso, Italy. Both mushroom and red chicory juices were prepared by homogenization of frozen fungi and fresh vegetable. To do this, aliquots (400 g) of frozen fungi or aliquots (500 g) of fresh red chicory, were homogenized for 2 min and centrifuged for 10 min at 8,000 × g. Both juices, after separation from the solid part, were filtered on paper filter and then sterilized by ultrafiltration. The LMM fractions (< 5,000 Daltons) of both shiitake mushroom and red chicory were prepared by ultrafiltration of the respective crude homogenate using the Vivaflow 200 system (Vivascience AG, Hannover, Germany) equipped with a membrane 5,000 MWCO PES for ultradiafiltration, as described elsewhere [8]. About 70 and 50% (w/w) of the components originally present in the crude mushroom and chicory homogenates, respectively, were detected in the ultradiafiltrates. The ultradiafiltrates were sterilized using a 0.20 μm pore size membrane (Vivascience), then freeze-dried and stored up to 3 months at −80°C. Immediately before use, a sample was rehydrated with sterile distilled water to obtain a 10x solution and kept at 4°C for no longer than a week. The 1x concentration of the LMM fraction after reconstitution represents the original concentration in the food.

### Evaluation of cell growth and viability

Optical density (O.D.) was measured at 640 nm wavelength with a Beckman mod. DU 530 spectrophotometer. Total bacteria were counted as cell particles with a Coulter Counter mod ZBI (Coulter Scientific) equipped with a 30 μm capillary. Viable cells were determined as colony forming units (CFU) per ml of culture. Suitable dilutions in sterile saline solution of the untreated and treated cultures were plated on BHIA and plates incubated at 37°C for 24 hours in the capnophylic atmosphere.

### Evaluation of DNA, RNA and protein synthesis

Aliquots of an exponentially growing culture (150 μl, O.D. 0.2 unit) were placed in the wells of a microtitre plate together with 5 μCi of [^3^H] thymidine (spec. act. >10 Ci/mmol) or [^3^H] uridine (spec. act. >20 Ci/mmol) or [^3^H] leucine (spec. act. 50 Ci/mmol, PerkinElmer Life and Analytical Science, Boston, MA, USA). In some wells suitable dilutions of the test compounds were added. Each determination was performed in triplicate. Microtiter plates were incubated at 37°C in the capnophylic atmosphere for 60 min. At the end of this time period the culture was overlaid on a Millipore GF/C glass fibre paper soaked in advance with 10% trichloroacetic acid (TCA) and plunged in a cold 10% TCA solution. Filters were then washed three times with cold TCA and, finally, twice with acetone. Radioactivity was determined with a Beckman LS 6500 liquid scintillation counter. Three distinct identical experiments were performed.

### Scanning electron microscopy (SEM)

SEM was performed within a week of fixation of the samples. Glutaraldehyde-fixed samples from both planktonic and biofilm cultures were washed three times with phosphate buffer followed by three rinses in distilled water. Specimens were then serially dehydrated in ethanol and subjected to freeze drying, mounted on metal stubs and sputter-coated with gold. Each specimen was viewed with an ESEM FEG XL30 electron microscope (Fei-Philips) at magnifications of x5,000 to x40,000. [12]. Cell length measurement was performed on 150 bacteria per sample at x10,000 magnification by Image J software (http://rsbweb.nih.gov/ij/index.html). Data handling and curve fitting with a bimodal Gaussian distribution were performed by Microcal Origin 6.0 (Northampton, MA, USA).

### Cytoplasmic and membrane protein extraction for proteome analysis

One litre of an exponentially growing culture of *S*. *mutans* UA159 (O.D. 0.8 units) and one litre of a same culture treated for 60 min with 2x mushroom extract were rapidly chilled in ice and immediately centrifuged in a Sorvall RC-5B refrigerated centrifuge with a GS-3 rotor (5,000 ×g for 10 min). Pellets were washed three times with cold 50 mM phosphate buffer (ph 7.2) and cells fragmented with glass beads (0.10 mm) in a B. Braun Cell Disruptor (B. Braun, Melsungen, Germany) under a continuous CO_2_ flow to keep the vial refrigerated. Unbroken cells were removed by centrifugation at 2,500 ×g for 10 min at 4°C and supernatant was centrifuged at 30,000 ×g with a type 40 rotor in a Beckman Optima LE-80 K ultracentrifuge (Beckman Coulter, Inc.) for 60 min. Supernatant (cytoplasmic fraction) was removed and pellet (cytoplasmic membrane and cell wall) washed with phosphate buffer and centrifuged as above three times. Cytoplasmic fractions of treated and untreated *S*. *mutans* were precipitated with 15% of trichloroacetic acid (TCA) in ice and resuspended in 1 ml of a solution containing 1 M Tris, 4 M NaCl, 2% cetyl-trimethylammonium bromide (CTAB) and 1× protease inhibitor cocktail tablet (Complete, Mini; La Roche). Proteins were purified as previously described by Polati *et al*. [13]. Briefly, nine volumes of double distilled water were added to the supernatants and samples were centrifuged 20,000xg for 10 min to remove DNA and polysaccharides. Proteins were then precipitated by addition of four volumes of cold acetone and purified by centrifugation at 20 000 × g for 10 min, and by washing the precipitates twice with absolute ethanol and twice with acetone. As far as the membrane proteins were concerned, the extraction from treated and untreated cells was carried out according to Zuobi-Hasona *et al*. [14]. Membrane pellet was resuspended in 500 μl (NH_4_)HCO_3_ 50 mM and 3 ml of a 2:1 (v/v) trifluoroethanol/chloroform mixture were added to the sample which was maintained at 0°C for 1 h vortexing every 5 min for 10 sec each. The mixture was centrifuged at 10,000xg for 5 min and the insoluble interphase was removed, and the remaining mixture was dried using an Eppendorf Vacufuge vacuum centrifuge (Eppendorf srl, Milan, Italy). The residue was suspended in a solubilisation buffer consisting of 7 M urea, 2 M tiourea, 4% CHAPS, 0,5% ASB14, 2% Triton X-100, pH 3–10 Ampholyte (Fluka, Buchs SG, Switzerland). Proteins were then precipitated by addition of acetone/methanol 8:1 (v/v) and purified by centrifugation at 20 000× g for 10 min. All the resulting protein pellets, both from cytoplasmic and from membrane fractions, were solubilised with a solution containing 7 M urea, 2 M thiourea, 3% CHAPS, 20 mM Tris. Then 1% pH 3–10 Ampholyte was added and samples were centrifuged for 30 min to remove residual contaminants complexed with ampholytes. Samples were incubated with 5 mM tributyl phosphine and 20 mM acrylamide for 60 min at room temperature to reduce protein disulphide bonds and alkylate the cysteine thiolic groups. The reaction was blocked by the addition of 10 mM DTT Q and samples were stored at −80°C. Protein concentration was evaluated with Bradford assay [15].

### Two-dimensional gel electrophoresis (2-DE)

Protein fractionation by 2-DE was performed by rehydrating seven cm long, pH 3–10 immobilized pH gradient strips (IPG; Bio-Rad Laboratories, Hercules, CA, USA) for 6 h with 150 μL of the sample solution containing 800 μg of total protein from *S*. *mutans* cells. Isoelectric focusing (IEF) was carried out with a Protean IEF Cell (Biorad), with a low initial voltage and then by applying a voltage gradient up to 5,000 V with a limiting current of 50 μA/strip and the temperature was set at 20°C. For the second dimension, the IPGs strips were equilibrated for 26 min by rocking in a solution of 6 M urea, 2% SDS, 20% glycerol, 375 mM Tris–HCl, pH 8.8. The IPG strips were then laid on 10-20% gradient SDS-PAGE with 0.5% agarose in the cathode buffer (192 mM glycine, 0.1% SDS and Tris–HCl to pH 8.3). The electrophoretic run was performed by setting a current of 5 mA for each gel for 30 min, then 10 mA/gel for 1 h, and 20 mA/gel until the end of the run. During the run the temperature was set at 11°C. The protein spots were revealed with Sypro Ruby stain (Life Technologies Corporation).

### Image analysis

The image analysis of the 2D gels replicates was performed by PDQuest software (Bio-Rad), version 7.3. Each gel was analyzed for spot detection, background subtraction and protein spot OD intensity quantification. The gel image showing the higher number of spots and the best protein pattern was chosen as a reference template, and spots in a standard gel were then matched across all gels. Spot quantity values were normalized in each gel dividing the raw quantity of each spot by the total quantity of all the spots included in the standard gel. Two distinct differential analyses were performed, one for cytoplasmic proteins and one for membrane fractions. In both the experiments gels were divided in two separated groups (control and treated samples) and, for each protein spot, the average spot quantity value and its variance coefficient in each group were determined. A Student's test was performed in order to compare the two groups and identify sets of proteins that showed a statistically significant difference with a confidence level of 0.05.

### Peptide sequencing by nano HPLC-ESI-MS/MS

In gel digestion by trypsin was performed, according to Shevchenko *et al*. [16]. Peptide mixtures were separated by using a nanoflow-HPLC system (Agilent Technologies series 1200, Santa Clara, CA, USA). A sample volume of 10 μl was loaded by the autosampler onto a 0.3 mm fused silica pre-column (75 μm I.D.; 375 μm O.D.) at a flow rate of 10 μl/min. Sequential elution of peptides was accomplished by using a flow rate of 250 nl/min and a linear gradient from Solution A (2% acetonitrile; 0.1% formic acid) to 50% of Solution B (98% acetonitrile; 0.1% formic acid) in 40 minutes over the pre-column in-line with a homemade 15 cm resolving column (75 μm I.D.; 375 μm O.D.; Zorbax 300-SB C18, Agilent Technologies). Peptides were eluted directly into an ion Trap (model Esquire 6000, Bruker-Daltonics, Bremen, Germany). Capillary voltage was 1.5-2 kV and a dry gas flow rate of 10 L/min was used with a temperature of 230°C. The scan range used was from 300 to 1800 m/z. Protein identification was performed by searching in the National Center for Biotechnology Information non-redundant database (NCBInr) using the Mascot program (http://www.matrixscience.com). The following parameters were adopted for database searches: complete carbamidomethylation of cysteines and partial oxidation of methionines, peptide Mass Tolerance ± 1.2 Da, Fragment Mass Tolerance ± 0.9 Da, missed cleavages 2. For positive identification, the score of the result of [−10 × Log(P)] had to be over the significance threshold level (p < 0.05).

### Chemicals and reagents

Unless otherwise specified, chemicals and reagents were purchased from Sigma Aldrich, St. Louis, MO, USA).

## Results

### Effects of LMM fractions of both mushroom and chicory extracts on cell growth and viability of *S. mutans*

**Figure 1:**
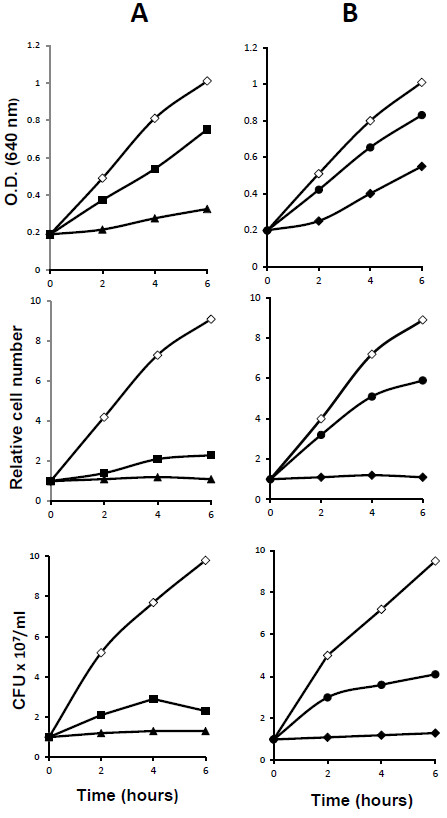
**Effects on cell mass, cell number, and cell viability of *S. mutans *treated with different concentration of LMM fraction of mushroom (column A) and chicory (column B).** Column A symbols: ( ◊ ) untreated control, ( ■ ) 1x, ( ▲ ) 2x; column B symbols: ( ◊ ) untreated control, 2x ( ● ), 6x ( ♦ ) concentration.

Different concentrations of LMM fraction of either mushroom or chicory were tested on cell growth of *S*. *mutans*. Figure 1 shows the effects of different concentrations on increase in optical density, cell particle number, and cell viability of *S*. *mutans*. As far as the LMM fraction of mushroom extract concerned (Figure 1, column A), the concentration of 2x was the minimum capable of inhibiting cell division of *S*. *mutans* as evaluated by cell particle counts, while 1x allowed a slight increase of the total cell number over the experiment and corresponded to two doublings. On the contrary, both concentrations only partially reduced O.D. increase in a dose-dependent manner. Evaluation of cell viability indicated a bacteriostatic effect of the 2x concentration. Concentrations of LMM fraction of chicory extract were also tested in *S*. *mutans* (Figure 1, column B). As compared with LMM fraction of mushroom extract, the 2x concentration was endowed of very limited inhibitory activity. Only 6x concentration was capable of complete inhibition of cell division but without exerting bacterial killing. At this concentration a three fold increase in O.D. of the culture was observed.

### Effects of LMM fraction of mushroom and chicory extracts on macromolecular synthesis of *S. mutans*

DNA, RNA and protein synthesis of *S*. *mutans* were evaluated in the presence of the above reported active concentrations (i.e. the minimal inhibitory and a sub-inhibitory concentration). Table 1 summarises the results: strong inhibitory effect on DNA synthesis was observed during treatment with 2x or 6x concentration of mushroom and chicory extracts, respectively, with a residual synthesis lower than 10% of the untreated control. The 1x or 2x sub-inhibitory concentration of mushroom and chicory extracts, respectively, allowed roughly 24-36% DNA synthesis. RNA synthesis, albeit to a lesser extent compared with DNA synthesis, was reduced to roughly 20% of the control in the presence of the inhibitory concentrations and to 34-54% of the control in the presence of the sub-inhibitory concentration of both mushroom and chicory extracts, respectively. Protein synthesis was inhibited by roughly 50% in the presence of the inhibitory dose of both mushroom and chicory extracts and to a lesser extent (11-39%) by sub-inhibitory concentrations.

**Table 1:** **Macromolecular synthesis of *S. mutans *treated for 60 min. with different concentrations of LMM fractions of both mushroom and chicory extracts**

**LMM fraction**	**Concentration**	**Macromolecular synthesis**		
		**DNA**	**RNA**	**Protein**
mushroom	0	100 ^b^	100 ^b^	100 ^b^
	1x ^a^	24	34	61
	2x	8	21	49
chicory	0	100 ^c^	100 ^c^	100 ^c^
	2x ^a^	36	54	89
	6x	6	19	44

^a^ Subinhibitory dose.

^b^ Control values were: DNA, 287,085 cpm; RNA, 137,216; protein, 9,208.

^c^ Control values were: DNA, 252,718 cpm; RNA, 132,519; protein, 8,749.

### Morphological examination and cell size distribution of *S. mutans* treated with LMM fractions of mushroom and chicory extracts

**Figure 2:**
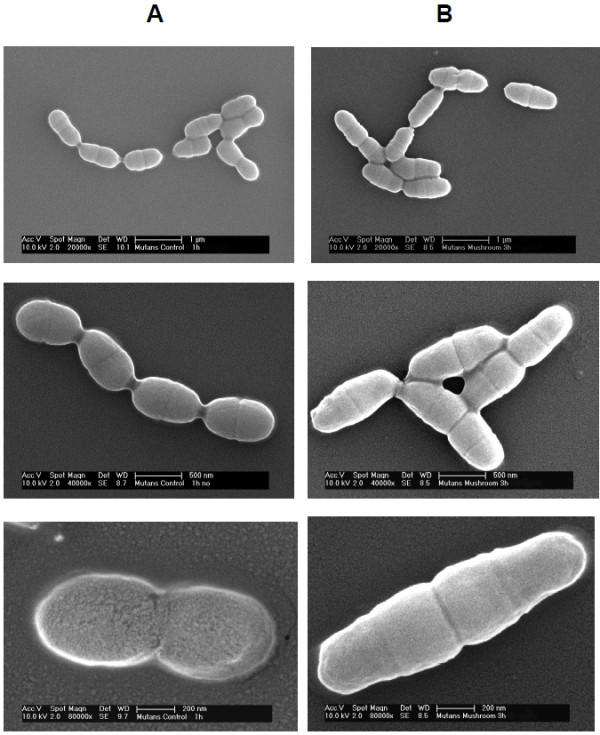
**Scanning electron microscopy of untreated *S. mutans *(column A) and after a three-hour treatment with 2x of LMM fraction of mushroom extract (column B) at different magnifications.**

**Figure 3:**
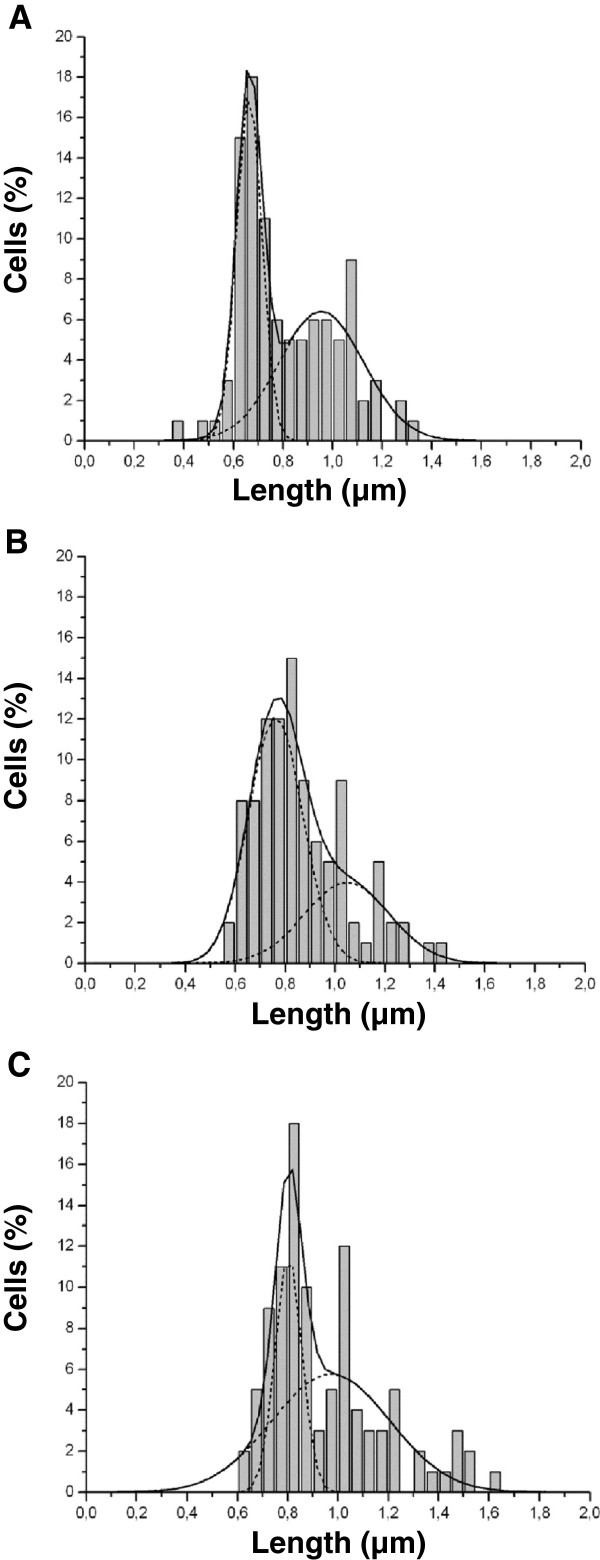
**Cell length distribution of *S. mutans *(A) and after a three-hour treatment with 1x (B) and 2x (C) LMM fraction of mushroom extract.** X_1_ (centre of the first Gaussian fit) values were 0.663 for image **A**, 0.761 for **B** and 0.806 μm for **C**; W_1_ (width at half maximum of the first Gaussian fit) values were 0.101, 0.218 and 0.126 μm for images **A**, **B** and **C**, respectively; X_2_ (centre of the second Gaussian fit) values were 0.955, 1.047 and 0.993 μm for images **A**, **B** and **C**, respectively; W_2_ (width at half maximum of the second Gaussian fit) values were 0.342, 0.342 and 0.45 μm for images **A**, **B** and **C**, respectively.

Bacteria treated as above were also collected for morphological analysis by either optical and scanning electron microscopy. Preliminary observation by optical microscopy showed the presence of elongated cells as a result of the treatment. Thus, we resorted to SEM analysis in order to precisely evaluate cell size and distribution. Figure 2 shows the appearance of *S*. *mutans* during treatment with 2x LMM fraction of the mushroom extract. Elongated cells with interrupted septa were seen after a three-hour treatment, while untreated control cells presented the typical streptococcal appearance as ovoidal cells. Similar morphological changes were observed during treatment with the 6x chicory extract as well as with the above reported sub-inhibitory concentration of both mushroom and chicory (data not shown). Table 2 shows the mean cell length with additional parameters of the treated cells in comparison with the untreated ones. Measures confirmed the morphological changes at the inhibitory concentration, however it is worth of note that elongation has been observed also in cells treated with sub-inhibitory concentrations. Furthermore, analysis of the size distribution of untreated control *S*. *mutans* cells showed that the characteristic Gaussian bimodal distribution, i.e. just divided single cells and doubled cells prior of dividing, as previously reported for streptococci, was no longer evident in treated cells as a result of global cell elongation (Figure 3).

**Table 2:** **Cell length measurement of *S. mutans *cells treated for three hours with different concentrations of LMM fractions of both mushroom or chicory extracts in comparison with control cells at the same time**

**Microorganism**	**Growth condition**	**Cell length (μm)**				
		**Mean**	**SD**	**Min. value**	**Max. value**	**Mode**
*S. mutans*	control	0.78	0.16	0.48	1.28	0.59
	Mushroom 1x ^a^	0.89	0.18	0.56	1.47	0.78
	Mushroom 2x	0.98	0.23	0.60	1.82	0.84
	Chicory 2x ^a^	0.86	0.18	0.55	1.46	0.75
	Chicory 6x	0.95	0.23	0.60	1.60	0.82

^a^ Subinhibitory dose.

**Figure 4:**
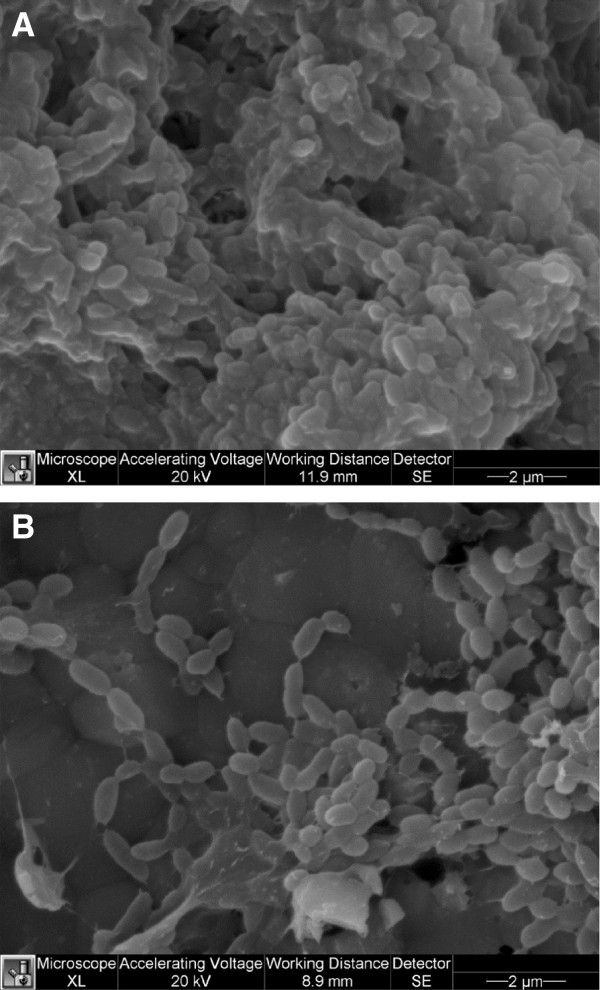
**Scanning electron microscopy of *S. mutans *grown in biofilm state (A) and after treatment with 2x LMM fraction of mushroom extract (B).** Magnification x12,000.

In a further set of experiments we evaluated the effects of the LMM fraction of mushroom extract on *S*. *mutans* biofilm architecture. Figure 4 shows that untreated control biofilm appeared as a compact structure with bacteria close to one another, while in treated biofilm several gaps were observed.

### Proteome analysis of *S. mutans* treated with LMM fraction of mushroom

**Figure 5:**
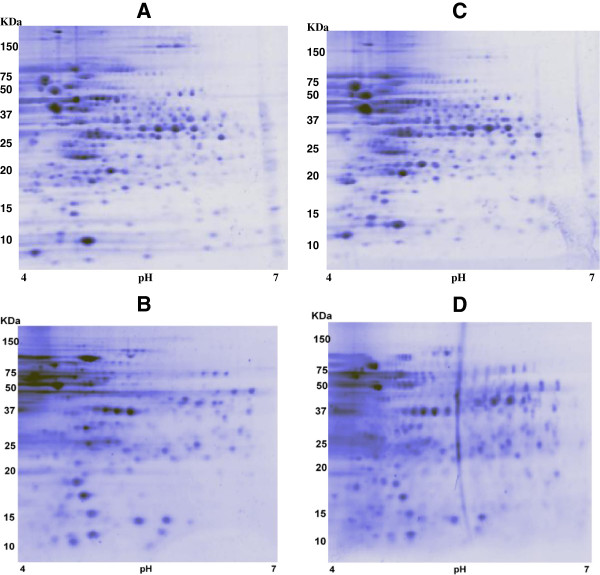
**2DE cytosol (A) and membrane (C) protein profile of *S. mutans *and after treatment with 2x LMM fraction of mushroom extract (B and D, respectively).**

Proteome analysis was conducted in both cytoplasmic and membrane fractions of *S*. *mutans*. Figure 5 shows a representative separation by 2D-PAGE of the cytosol or membrane proteins of an untreated and a treated *S*. *mutans* culture. Several dozen of protein spots that significantly either increased or lowered in treated cells in comparison with those corresponding of the untreated control, were identified after peptide sequencing by nano HPLC-ESI-MS/MS. Only proteins that quantitatively changed ±2 fold were considered significantly varied. Table 3 summarises results by grouping the vast majority of the 31 changed membrane proteins on the base of their physiological roles. Interestingly, all (eight) proteins involved in membrane transport significantly decreased, while several other proteins involved in sugar biosynthesis and metabolism, protein biosynthesis and folding, cell cycle and division, aminoacid biosynthesis and metabolism, or miscellanea were either up-regulated or down-regulated in treated *S*. *mutans* cells. Table 4 summarises results of the study of the cytosol fraction of either control or treated cells. A total of 20 proteins were analysed for their sequence but only one of them remained unidentified. Grouping on the base of their physiological roles have been achieved: 5/5 proteins involved in stress response (e.g. GroEL, GroES, DnaK, SodA, GshR) and 4/4 involved in cell redox homeostasis were all significantly up-regulated, while of the 6 proteins involved in sugar biosynthesis and metabolism 4 were down- and 2 up-regulated, and, finally, 2 proteins were up- and 2 were down-regulated of the 4 proteins involved in protein biosynthesis and folding.

**Table 3:** **Identification by nano HPLC-ESI-MS/MS of *S. mutans *membrane proteins differentially expressed when treated with 2x LMM fraction of mushroom extract**

**Protein name**	**Spot no.**	**Fold of variation**	**NCBI acc. #**	**Gene name**	**Theor. Mr. (Da)**	**Theor. pI**	**No. of peptides identified**	**Mascot score**^**a)**^	**Sequence coverage**^**b)**^**(%)**	**Molecular function**
***MEMBRANE TRANSPORT***										
F0F1 ATP synthase subunit delta	2202	- 2.27	gi|24379920	*atpH*	20436	5.22	9	528	51	ATPase activity
Putative ABC transporter, ATP-binding protein	5303	- 2.08	gi|24380277	*psaA*	26297	5.58	12	694	57	ATPase activity
Putative oligopeptide ABC transporter, ATP-binding protein	5601	- 2.38	gi|24378766	*oppD*	39147	5.47	15	680	56	ATPase activity
Putative sugar ABC transporter, ATP-binding protein	5804	- 7.32	gi|24379552	SMU_1120	55575	5.78	26	1197	62	ATPase activity
Putative amino acid ABC transporter, ATP-binding protein	6406	- 2.56	gi|24378949	SMU_461	27674	5.83	19	847	75	ATPase activity
Putative amino acid ABC transporter, ATP-binding protein	7303	- 9.09	gi|24379380	SMU_936	28340	5.90	2	118	10	ATPase activity
Putative phosphate ABC transporter, ATP-binding protein	7304	- 2.17	gi|24379566	*pstB*	30276	5.90	3	136	12	ATP binding
Multiple sugar-binding ABC transporter, ATP-binding protein	7702	- 2.94	gi|24379336	*msmK*	41938	5.92	14	579	39	ATPase activity
***SUGAR BIOSYNTHESIS AND METABOLISM***										
Putative glucose-1-phosphate thymidyltransferase	2402	- 2.50	gi|24379858	*rmlA*	32275	4.90	16	917	53	Tranferase activity
Pyruvate kinase	4101	+ 2.76	gi|24379618	*pykF*	54333	5.09	2	70	5	Kinase activity
Glyceraldehyde-3-phosphate dehydrogenase	4601	+ 2.28	gi|24378857	*gapC*	36046	5.71	17	725	44	Oxidoreductase activity
Galactose 6-P isomerase	6102	+ 3.01	gi|153674	*lacA*	19025	5.80	2	84	15	Isomerase activity
Catabolite control protein A	8501	- 6.14	gi|2155300	*ccpA*	36590	7.08	3	124	9	DNA binding
***PROTEIN BIOSYNTHESIS AND FOLDING***										
60 kDa chaperonin	5102	+ 2.19	gi|13898679	*cpn60*	19827	4.53	2	87	14	Protein binding
Phenylalanyl-tRNA synthetase subunit alpha	6603	- 7.83	gi|24379902	*pheS*	39271	5.87	14	420	34	Nucleotide binding
30S ribosomal protein S8	9103	+ 2.24	gi|24380355	*rpsH*	14684	9.10	7	411	64	RNA binding
***CELL CYCLE AND DIVISION***										
Putative Hit-like protein	2102	+ 2.07	gi|24378905	SMU_412c	15560	5.13	3	139	30	Unknown
Hypothetical protein SMU.471	4002	- 2.04	gi|24378958	SMU471	12972	5.37	5	255	42	Methylase activity
UDP-N-acetylglucosamine 1-carboxyvinyltransferase 1	5702	+ 2.30	gi|24379914	*murA1*	45588	5.65	10	422	30	Tranferase activity
***AMINOACIDS BIOSYNTHESIS AND METABOLISM***										
Uridylate kinase	3302	+ 4.08	gi|24380005	*pyrH*	26315	5.48	9	460	52	Kinase activity
Phospho-2-dehydro-3-deoxyheptonate aldolase	4505	+ 2.60	gi|24380198	*aroG*	39072	6.02	8	338	29	Lyase activity
Shikimate 5-dehydrogenase	5401	+ 2.36	gi|24379237	*aroE*	31791	6.19	2	106	11	Oxidoreductase activity
Putative D-3-phosphoglycerate dehydrogenase	5604	- 3.33	gi|24380031	*serA*	42771	5.56	14	815	51	Oxidoreductase activity
***MISCELLANEOUS***										
Putative dihydrolipoamide dehydrogenase	3603	+ 4.23	gi|24378648	*adhD*	61708	4.88	4	270	10	Oxidoreductase activity
Orotate phosphoribosyltransferase	4201	+ 2.38	gi|24379645	*pyrE*	22802	5.37	7	381	45	Glycosyltransferase activity
Putative exodeoxyribonuclease III	6403	- 2.56	gi|24380027	*smnA*	31393	5.72	11	558	44	DNA binding
(3R)-hydroxymyristoyl-ACP dehydratase	7403	- 2.08	gi|24380109	*fabZ*	15361	8.83	3	150	21	Lyase activity
Not identified	5301	+ 4.52								
Not identified	8205	+ 2.59								
Not identified	8401	- 4.35								
Not identified	8506	+ 3.30								

^a)^ Score is –10^6^log(*p*), where *p* is the probability that the observed match is a random event, based on the NCBI database using the MASCOT searching program as MS/MS data.

^b)^ Amino acid sequence coverage for the identified protein.

**Table 4:** **Identification by nano HPLC-ESI-MS/MS of *S. mutans *cytosol proteins differentially expressed when treated with 2x LMM fraction of mushroom extract**

**Protein name**	**Spot no.**	**Fold of variation**	**NCBI acc. #**	**Gene name**	**Theor. Mr. (Da)**	**Theor. pI**	**No. of peptides identified**	**Mascot score**^**a)**^	**Sequence coverage**^**b)**^**(%)**	**Molecular function**
***STRESS RESPONSE***										
60 kDa chaperonin	1605	+ 2.17	gi|21666296	*groEL*	56428	4.68	39	2392	78	Protein binding
Molecular chaperone dnaK	1706	+ 2.29	gi|24378606	*dnaK*	65246	4.58	28	1265	51	Protein binding
10 kDa chaperonin	3001	+ 3.25	gi|21666295	*groES*	10064	4.93	4	273	32	Protein binding
Superoxide dismutase	4108	+ 2.60	gi|24379102	*sodA*	22611	4.99	9	521	56	Ion binding
Glutathione reductase	6607	+ 3.89	gi|4587160	*gshR*	48955	5.40	3	143	7	Oxidoreductase activity
***SUGAR BIOSYNTHESIS AND METABOLISM***										
Glucose-1-phosphate adenylyltransferase	2501	- 3.57	gi|92081399	*glgC*	41906	4.72	13	680	38	Transferase activity
Phosphopyruvate hydratase	2806	- 2.56	gi|24379669	*eno*	46829	4.67	14	771	40	Lyase activity
Putative phosphoglucomutase	3703	- 12.50	gi|24379514	*pgm*	63056	4.88	33	1783	69	Transferase activity
Tagatose 1,6-diphosphate aldolase 1	4303	- 2.17	gi|153676	*lacD1*	36468	4.96	11	462	31	Lyase activity
Putative N-acetylglucosamine-6-phosphate isomerase	6205	+ 2.43	gi|24379109	SmuNN2025_1351	25457	5.33	7	330	37	Deaminase activity
Fructose bi-phosphate aldolase	8001	+ 5.24	gi|4322370	*fba*	7447	8.85	3	115	30	Lyase activity
***PROTEIN BIOSYNTHESIS AND FOLDING***										
Elongation factor Tu	1603	- 2.44	gi|24379182	*tuf*	43891	4.84	25	1257	72	GTPase activity
Trigger factor	1701	+ 2.06	gi|24378614	*tig*	47457	4.46	20	918	51	Isomerase activity
50S ribosomal protein L4	3101	+ 2.18	gi|254998187	SmuNN2025_1762	22215	9.76	6	313	39	RNA binding
Elongation factor Ts	3503	- 2.38	gi|24380373	*tsf*	37695	4.91	22	1218	74	GTP binding
***CELL REDOX HOMEOSTASIS***										
Thiol peroxidase	0007	+ 4.33	gi|254997527	*tpx*	17541	4.56	8	450	34	Oxidoreductase activity
Alkyl hydroperoxide reductase	1107	+ 12.60	gi|24379223	*ahpC*	20465	4.57	6	284	34	Oxidoreductase activity
Putative bacterocin transport accessory protein, Bta	5002	+ 2.71	gi|24380154	SMU_1788c	12601	5.21	2	85	18	Oxidoreductase activity
Putative glutathione S-transferase	8202	+ 5.64	gi|24379712	SMU_1296	29973	5.74	5	243	16	Transferase activity
Not identified	8007	- 3.57								

^a)^ Score is –10^6^log(*p*), where *p* is the probability that the observed match is a random event, based on the NCBI database using the MASCOT searching program as MS/MS data.

^b)^ Amino acid sequence coverage for the identified protein.

## Discussion

Although foods have to be considered deleterious for oral health in that capable of sustaining growth of oral bacteria (e.g. sucrose-rich foods and dental caries), however a large number of scientific reports indicates that several commonly consumed foods and beverages of natural origin contain substances with a beneficial impact on oral health [5-7,17]. The healthy effects are attributed mainly to the polyphenol fraction which is present in a large number of vegetables [7]. These compounds have been shown to have antimicrobial, antiadhesive, antiplaque activities, thus the consumption of these foods and beverages could be encouraged in order to improve oral hygiene. Epidemiological studies have shown that people consuming foods rich in bioactive compounds have an improved oral health (e.g. tea drinkers have a less risk of dental caries) [5]. Furthermore, attempts to formulate active natural compounds as anticaries and/or antigingivitis oral rinses for daily oral hygiene have shown encouraging results, although a limited number of pilot clinical trials have been performed [5,6,18-22]. Among the numerous foods and drinks tested, the LMM fraction of both shiitake mushroom and red chicory aqueous extract has been previously demonstrated to be endowed of several antimicrobial features that render them of special interest as components of products for daily oral hygiene [8-10,21,22]. In this report we have evaluated the possible mode of action of these extracts on growth, morphology and physiology of *S*. *mutans*, the etiological agent of human dental caries. Of the two extracts tested, the best antimicrobial activity has been displayed by the LMM mushroom extract with a minimal inhibitory concentration corresponding to 2x the natural concentration of the vegetable, while the LMM fraction of chicory extract inhibited growth only at 6x concentration. Inhibition of cell division resulted in bacteriostatic effect in both cases. At the minimal inhibitory concentration of both extracts DNA synthesis was the main macromolecular synthesis inhibited, resulting in a residual synthesis less than 10%, RNA synthesis, although strongly inhibited, was less than that of DNA and, finally protein synthesis was inhibited only by roughly 50%. The residual RNA synthesis and the partial inhibition of protein synthesis is compatible with the observed significant increase in O.D as result of cell mass increase. The increase in these parameters is, in turn, linked to the morphological alteration observed by electron microscopy with transition from cocci or slightly elongated cocci of the untreated control to elongated rods as the consequence of septum synthesis inhibition as effect of the treatment. Analysis of cell size distribution confirms this statement: the typical bimodal distribution of the streptococcal growing control was shifted to higher values when cells elongated. These results are in agreement with the previous observations of the typical mode of cell wall growth and division of streptococci and enterococci [23,24].

The fact that tested natural extracts act by blocking cell division after primary inhibition of DNA synthesis is reminiscent of the chemotherapeutic agents included in the quinolone family [25]. Furthermore, the peculiar response of the macromolecular synthesis and the characteristic morphogenetic effects as a result of the treatment with either mushroom or chicory extracts have also been observed, similarly to quinolones, at sub-inhibitory doses [25]. The morphogenetic effects induced by mushroom or chicory treatment on *S*. *mutans* cells are indeed similar to those observed in streptococcal thermo-sensitive division mutants when incubated at non-permissive temperature or exposed to both sub- and inhibitory doses of β-lactam antibiotics [26]. These considerations as a whole lend further support to the hypothesis that the mode of action is comparable to that of antimicrobial families such as quinolones and β-lactams, thus, an antibiotic-like mode of action is suggested for these compounds [25,27,28].

Analysis of bacterial proteome conducted on separated cytoplasmic fraction from membrane fraction of mushroom treated vs. untreated *S*. *mutans* cells has showed that eight proteins involved in membrane transport significantly decreased and seven of these were ABC transporters. ABC systems can be divided into three functional categories: (i) importers of nutrients, (ii) exporters including toxins and (iii) members involved in translation of mRNA and DNA repair [29]. Interestingly, we have observed that transport of aminoacids, sugars and phosphate is negatively affected. This, in turns, may cause modification of both sugar and amino acid biosynthesis and metabolism with about half of the involved proteins up-regulated and half down-regulated. Quinolones treatment has been shown to induce expression of ABC efflux system PatA/Pat B in *Streptococcus pneumoniae* in order to extrude quinolone molecules from the bacteria cytoplasm [30]. Furthermore the overexpression of this ABC efflux system is associated whit *S*. *pneumoniae* resistance against quinolones [31]. Our study has not evidenced modification in efflux system during the mushroom treatment, thus suggesting that this cannot represent a biological base for a next acquisition of resistance against the mushroom extract. Protein biosynthesis and folding changes may be consistent with the observed protein synthesis persistence after cell division inhibition. In particular, elongation factors Tu and Ts are down-regulated. EF-Tu is activated upon GTP binding and forms a tertiary complex with aminoacylated elongator tRNAs in order to decode the genetic information. This last event triggers GTP hydrolysis and EF-Tu is released from ribosome. EF-Tu is then recycled into the active GTP form by the nucleotide-exchange factor EF-Ts [32]. Down-regulation of EF-Tu and EF-Ts is consistent with 50% global protein synthesis inhibition as result of treatment.

The cell stress originated during treatment is documented by the significant increase of stress response proteins such as DnaK, SodA, GroES, GroEL and GshR. The heat shock chaperon proteins DnaK, GroEL and GroES bind partially unfolded proteins [33]. These molecular chaperones bind transiently and noncovalently to nascent polypeptides and unfolded or unassembled proteins aiding the protein biogenesis. Superoxide dismutase SodA dismutases superoxide anions which are normally produced within cells and which are toxic to biological systems. Glutatione reductase GshR is important for aerobic growth in order to protect *S*. *mutans* cells from oxidative stress [34]. Similarly, several proteins involved in cell redox homeostasis are highly overexpressed as effect of mushroom extract treatment. Redox reactions are central to both anabolic and catabolic metabolism and, thus, vital to all organisms. Various molecular sensors continually monitor both internal and external environments adjusting processes involved in maintaining redox homeostasis. In response to redox imbalance, as results of several factors including an increase in oxidation reactions (oxidative stress), new metabolic pathways are initiated and/or systems that protect the cell from further damage are induced [35]. Thus, results from *S*. *mutans* proteome analysis, in their whole, are consistent with cell physiology changes in response to mushroom treatment.

## Conclusions

This report has shown an antibiotic-like mode of action of mushroom and chicory extracts as demonstrated by induced morphogenetic effects and inhibition of specific macromolecular synthesis. This feature as well as the safe use of this extract as result of its natural origin render the LMM both mushroom and chicory extracts suitable for the formulation into products for daily oral hygiene such as mouthwashes or toothpastes. Furthermore, encouraging the consumption of this food as well as enriching foods with this bioactive extract may be of great interest particularly in developing countries where economic conditions discourage the use of commercially available products for regular oral hygiene.

## Competing interests

The authors declared that they have no competing interest.

## Authors’ contributions

CS and PC equally contributed in experimental design, undertook data analysis and interpretation, and drafted the manuscript. AM, AB, and GB, carried out basic microbiology experiments. AM carried out 2DE, peptide sequencing by nano HPLC-ESI-MS/MS and data analysis. FT and IC performed SEM analysis and cell size distribution. AP carried out fractionation of plant extracts. CP, EZ, PL, IO, DAS, JP, and MW are part of the Nutrident consortium and aided in the general experimental design. All authors approved the final manuscript.

## Pre-publication history

The pre-publication history for this paper can be accessed here:

http://www.biomedcentral.com/1472-6882/13/117/prepub
